# Deciphering Molecular Mechanism of the Neuropharmacological Action of Fucosterol through Integrated System Pharmacology and *In Silico* Analysis

**DOI:** 10.3390/md17110639

**Published:** 2019-11-13

**Authors:** Md. Abdul Hannan, Raju Dash, Abdullah Al Mamun Sohag, Il Soo Moon

**Affiliations:** 1 Department of Anatomy, Dongguk University College of Medicine, Gyeongju 38066, Korea; hannanbau@gmail.com (M.A.H.); rajudash.bgctub@gmail.com (R.D.); 2 Department of Biochemistry and Molecular Biology, Bangladesh Agricultural University, Mymensingh 2202, Bangladesh; sohag2010bmb.sust@gmail.com

**Keywords:** Alzheimer’s disease, fucosterol, molecular simulation, network pharmacology, neurodegeneration, Parkinson’s disease

## Abstract

Fucosterol is an algae-derived unique phytosterol having several medicinal properties, including antioxidant, anti-inflammatory, anticholinesterase, neuroprotective, and so on. Accumulated evidence suggests a therapeutic promise of fucosterol in neurodegeneration; however, the in-depth pharmacological mechanism of its neuroprotection is poorly understood. Here, we employed system pharmacology and *in silico* analysis to elucidate the underlying mechanism of neuropharmacological action of fucosterol against neurodegenerative disorders (NDD). Network pharmacology revealed that fucosterol targets signaling molecules, receptors, enzymes, transporters, transcription factors, cytoskeletal, and various other proteins of cellular pathways, including tumor necrosis factor (TNF), mitogen-activated protein kinase (MAPK), phosphatidylinositol 3-kinase/protein kinase B (PI3K/Akt), neurotrophin, and toll-like receptor (TLR) signaling, which are intimately associated with neuronal survival, immune response, and inflammation. Moreover, the molecular simulation study further verified that fucosterol exhibited a significant binding affinity to some of the vital targets, including liver X-receptor-beta (LXR-β), glucocorticoid receptor (GR), tropomyosin receptor kinase B (TrkB), toll-like receptor 2/4 (TLR2/4), and *β*-secretase (BACE1), which are the crucial regulators of molecular and cellular processes associated with NDD. Together, the present system pharmacology and *in silico* findings demonstrate that fucosterol might play a significant role in modulating NDD-pathobiology, supporting its therapeutic application for the prevention and treatment of NDD.

## 1. Introduction

Neurodegenerative disorders (NDD), such as Alzheimer’s disease (AD), Parkinson’s disease (PD), and Huntington’s disease (HD) are the major causes of dementia worldwide. Although the exact mechanism is unclear, inflammation and oxidative stress might play pivotal roles in the pathogenesis of NDD [1]. The cellular processes, particularly NF-κB signaling mediated cell survival and Nrf2/KEAP1 signaling mediated antioxidant defense, are severely compromised in NDD. The upstream signaling pathways, including PI3K/Akt, MAPK, TNF, and TLR signaling, largely regulate inflammation and oxidative stress-related signaling systems. Therefore, targeting these signaling pathways might offer potential disease-modifying therapeutic strategies against NDD.

Phytosterols are important molecules not only for their structural and signaling functions in the host plants [2] but also for their diverse applications in medical science [3]. The highly reputed therapeutic activity of phytosterols lies in their cholesterol-lowering potential [4]. Moreover, phytosterols have shown an array of pharmacological properties ranging from antioxidation to anti-inflammation to neuroprotection [5]. Fucosterol is an algal sterol, which is isolated mostly from brown algae, indicating its abundance in this particular algal class. Evidence from the literatures supports its various biological activities, including antioxidant [6,7,8,9], anti-inflammatory [7,8,10,11,12,13], anticancer [14,15], antidiabetic [16], cholesterol homeostasis [17], cholesterol-lowering [18], antihyperlipidemic [19], hepatoprotective [6,20,21], immunostimulatory [22], antimicrobial [23], antiobesity [24,25], and antidepressant [26] potentials. Moreover, fucosterol exhibits inhibitory activity against acetyl- and butyryl-cholinesterase (AChE and BChE) [27,28] and β-secretase (an enzyme responsible for Aβ production, which is related to Alzheimer’s disease) [29]. Fucosterol also attenuated Aβ-induced neuronal death [27,30] and ameliorated cognitive impairment in the Aβ-induced neurotoxicity model of adult rats [30]. With this evidence, we anticipate that fucosterol possesses therapeutic promise against NDD, particularly AD.

The system biology approach combined with *in silico* analysis offers an efficient computation tool that can better explain how a bioactive molecule interacts with the signal molecules of various cellular pathways and contribute in the therapy of multifactorial disease like NDD. Here, we employed integrated system pharmacology and *in silico* analysis to explain the pharmacological mechanism of fucosterol against NDD. First, through multiple bioinformatics tools, we demonstrate that fucosterol highly interacted with signaling pathways related to the development and survival of neuron, inflammation and immune response. Next, we employed *in silico* analysis to verify the biophysical interaction of fucosterol with some potential targets. The present study elucidates the underlying mechanism of molecular neuropharmacology of fucosterol and establishes a basis in favor of its therapeutic application against neurodegeneration.

## 2. Results

### 2.1. ADME/T Properties of Fucosterol

**Figure 1:**
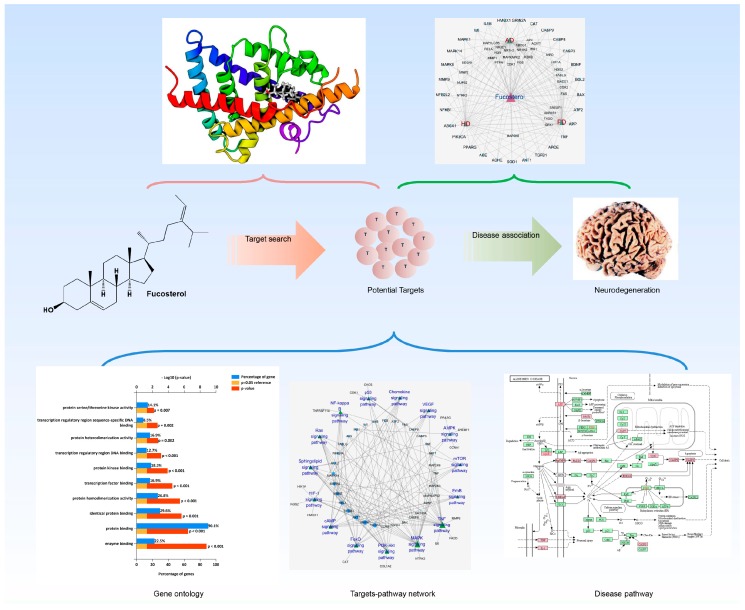
Schematics on the system pharmacology overview deciphering neuropharmacological action mechanism of fucosterol against neurodegeneration.

A diagrammatic overview displaying the different steps of network pharmacology was illustrated in Figure 1. We first evaluated the drug-like properties of fucosterol using QikProp ADME/T (absorption, distribution, metabolism, and excretion/transport) prediction tool. Fucosterol exhibited drug-like attributes (Table S1) and more likely to be orally available as it maximally (three out of four, and two out of three, respectively) obeyed the Lipinski’s rule of five [31] (mol_MW < 500, donor HB ≤ 5, and acceptor HB ≤ 10) and Jorgensen’s rule of three [32] (QPlogS > −5.7 and QPPCaco > 22 nm/s). Moreover, the predicted brain/blood partition coefficient (QPlogBB) of fucosterol was −0.3, which falls within the recommended range (−3.0–1.2), indicating that it can cross the blood–brain barrier.

### 2.2. Target Fishing

A total of 72 potential targets of fucosterol were retrieved from Pathway Assembly from Literature Mining-an Information Search Tool (PALM-IST) database (Table S2) and validated through literature scanning in the PubMed database. Targets having a direct connection with brain functionality were primarily considered.

### 2.3. Network Building

#### 2.3.1. Fucosterol–Target–Target (F–T–T) Network

**Figure 2:**
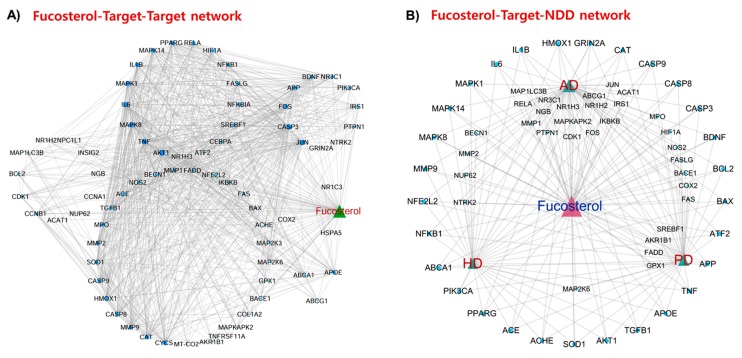
Network analysis: Fucosterol–target–target (F–T–T) network (**A**) and fucosterol–target–neurodegenerative disorders (F–T–NDD) network (**B**). Circular nodes represent targets of fucosterol. Node size is proportional to its degree. Edges represent interaction of target proteins with fucosterol and NDD. AD, Alzheimer’s disease; PD, Parkinson’s disease; HD, Huntington‘s disease.

F–T–T network demonstrated the interaction between fucosterol and its targets as well among the targets and identified most interacted protein targets in the network (Figure 2A). This network revealed that *BDNF*, *APP*, *APOE*, *AKT1*, *PPARG*, *NFKB1*, *NFE2L2* (Nrf2), *CYSC*, *MMP9*, *MAPK1*, *SOD1*, *JUN*, *FOS*, *CAT*, *TNF*, *IL6*, *IL1B*, *NOS2*, *CASP3*, *CASP9*, and *HMOX1* are the dominant hub genes that participate in the major cellular pathways.

#### 2.3.2. Fucosterol–Target–Neurodegenerative Disorders (F–T–NDD) Network

F–T–NDD network presented the interaction of fucosterol with those targets that are associated with NDD. A total of 56, 41, and 34 targets, respectively, were associated with AD, PD, and HD (Figure 2B). Among the targets, *BDNF*, *BCL2*, *BAX*, *ATF2*, *APP*, *TNF*, *APOE*, *TGFB1*, *AKT1*, *SOD1*, *AChE*, *ACE*, *PPARG* (PPAR-γ), *PIK3CA*, *ABCA1*, *NFKB1*, *NFE2L2* (Nrf2), *MMP9*, *MAPK1*, *IL6*, *IL1B*, *HMOX1*, *GRIN2A* (GluN2A), *CAT*, *CASP3*, and *CASP9*, each was connected with NDD, indicating the involvement of these targets or others to their upstream signaling as a potential drug-target for fucosterol in the NDD treatment. Moreover, there are some other potential targets, including *NTRK2* (TrkB), NR1H2 (LXR-β), NR3C1 glucocorticoid receptor, (GR), and β-secretase, that are closely associated with pathways and cellular processes in NDD pathology.

### 2.4. Gene Ontology (GO) Analysis

**Figure 3:**
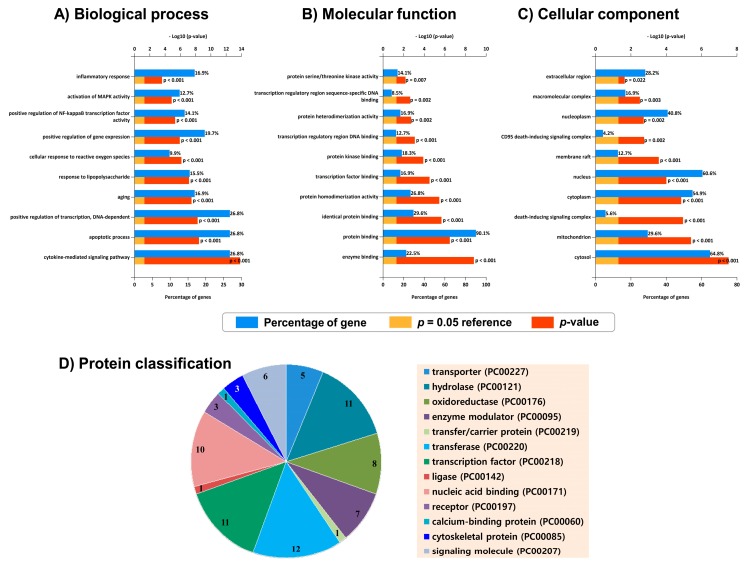
Bioinformatics analysis of fucosterol target genes. Gene ontology (GO) analysis by the DAVID annotation tool: Top 10 GO terms for biological processes (**A**), molecular functions (**B**), and cellular components (**C**) were displayed where the y-axis representing GO terms for the target genes, the upper x-axis showing *p* < 0.05 and the lower x-axis showing gene counts in terms of percentage. Panther classification categorized target proteins into 13 classes (**D**). The number within each subsection in the pie chart indicates the protein number in the given functional class.

Top 10 highly enriched GO terms under cellular component (CC), biological process (BP), and molecular function (MF) were displayed (Figure 3A–C). The CC term with the highest gene sequestration was the cytosol (64.8%), followed by the nucleus (60.6%). The highly enriched MF term was protein binding (90.1%). Overrepresented biological processes include cytokine-mediated signaling pathway, apoptotic process, transcription regulation, inflammatory response, aging, response to lipopolysaccharide, NF-κB activity, and cellular response to reactive oxygen species (ROS), which are closely associated with the NDD pathobiology, suggesting the possibility that fucosterol could intervene the disease progression through modulating these biological processes. Moreover, the target proteins were categorized into 13 different classes based on their cellular function, indicating their functional diversity (Figure 3D).

### 2.5. KEGG Pathways and Protein Targets Related to NDD Pathobiology

**Figure 4:**
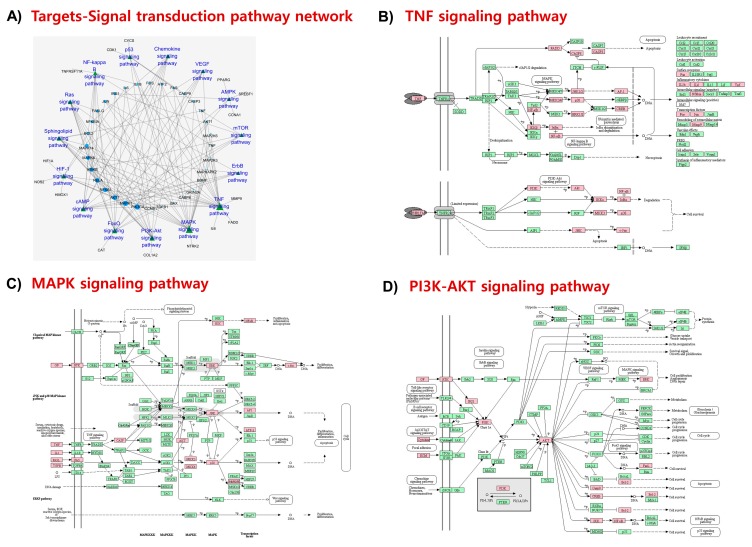
Signal transduction pathways in KEGG pathway analysis. A target-pathway network in the ‘signal transduction’ module (**A**). Top enriched pathways include TNF signaling pathway (**B**), MAPK signaling pathway (**C**) and PI3K-Akt signaling pathway (**D**). Fucosterol targets are highlighted in pink.

Enriched pathways were categorized into several modular systems, including signal transduction, nervous system, and immune system using Kyoto Encyclopedia of Genes and Genomes (KEGG) pathway annotation. A total of 15 signaling pathways were enriched (*p*-value < 0.01) in the “signal transduction” module (Figure 4A). Most overrepresented signal transduction pathways include TNF signaling (degree = 22; Figure 4B) and MAPK signaling (degree = 21; Figure 4C), which are involved in inflammatory response and neuronal survival, respectively. Other signaling systems involved in neuronal growth and survival include PI3K/Akt signaling (Figure 4D), FOXO signaling, and cAMP signaling. In addition, several other inflammation-related signaling pathways, including HIF-1 signaling, NF-κB signaling, and VEGF signaling, were also enriched (Figure 4A and Figure S1). LXR-β and GR, two of the fucosterol targeted proteins in our network pharmacology analysis (Figure 2), are involved in the regulation of the enriched inflammatory pathways. Moreover, TrkB, another fucosterol targeted protein, regulates the enriched signaling pathways involved in neuronal growth and survival. Therefore, LXR-β, GR, and TrkB will further be verified for their interaction with fucosterol by *in silico* analysis.

**Figure 5:**
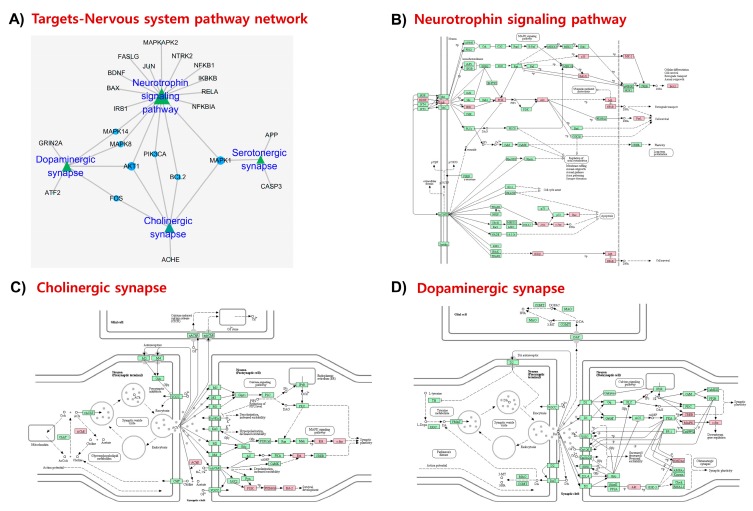
Nervous system pathways in KEGG pathway analysis. A target-pathway network in the ‘Nervous system’ module (**A**). Enriched pathways include neurotrophin signaling pathway (**B**), cholinergic synapse (**C**), and dopaminergic synapse (**D**). Fucosterol targets are highlighted in pink.

**Figure 6:**
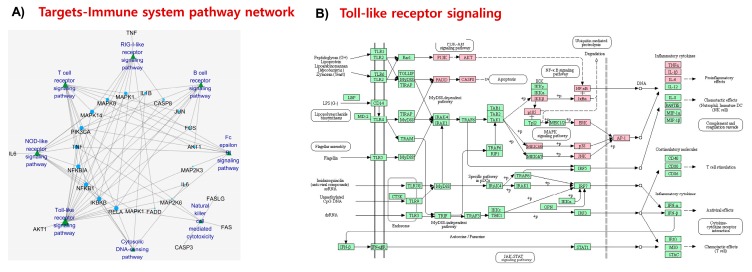
Immune system pathways in KEGG pathway analysis. A target-pathway network in the ‘Immune system’ module (**A**). Toll-like receptor signaling pathway (**B**) is the most over-represented immune-related pathway. Fucosterol targets are highlighted in pink.

Enriched nervous system-related pathways (Figure 5A) include neurotrophin signaling (degree = 17; Figure 5B), and cholinergic (Figure 5C), dopaminergic (Figure 5D), and serotonergic synapses, whose functional impairment is associated with NDD and related pathobiology. In addition, numerous immune-related signaling pathways (Figure 6A) including toll-like receptor signaling (degree = 18; Figure 6B), NOD-like receptor signaling (degree = 11), and T-cell receptor signaling (degree = 11) were enriched. Considering the significance of toll-like receptor signaling and the involvement of TLR2/4 in the immune signal recognition, the interaction of fucosterol with these receptor targets will further be validated through molecular docking.

**Figure 7:**
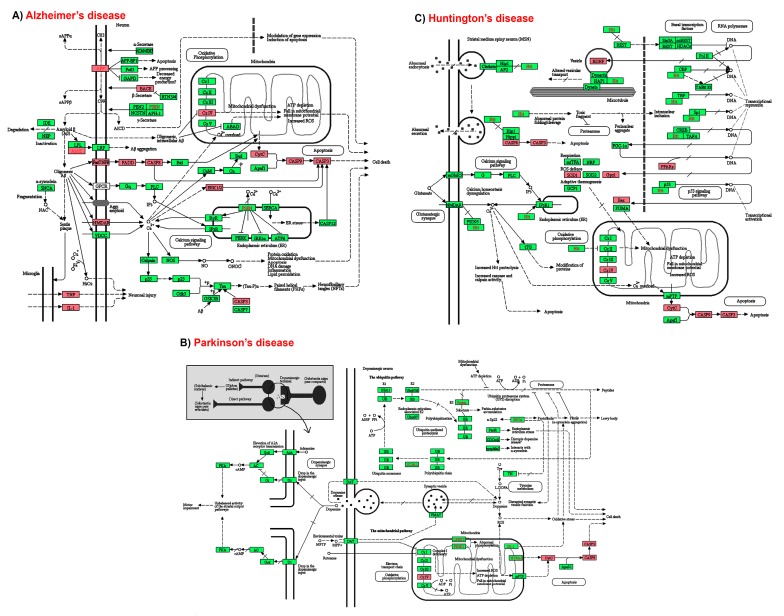
Pathways of neurodegenerative diseases (NDD) in KEGG pathway analysis. Pathways of three major NDD including Alzheimer’s (**A**), Parkinson’s (**B**), and Huntington’s (**C**) diseases linked many of the fucosterol targets, which are highlighted in pink.

Finally, NDD pathways were analyzed. In AD-pathway, a total of 14 target proteins were illustrated including those that are involved in amyloidogenesis (for example, BACE and APP), cholesterol homeostasis and Aβ-clearance (for example, ApoE), neuronal growth and survival (for example, Erk1/2 or *MAPK1*), synaptic plasticity (for example, GluN2A), inflammation (for example, *COX2*, *TNF*, and *IL1B*), and apoptosis (for example, *CASP3*, *CASP9*, *CASP8*, *CYCS*, *FAS*, and *FADD*) (Figure 7A). Among them, BACE interaction with fucosterol will further be validated through molecular docking because BACE inhibitor could be a potent anti-AD agent. The interaction of fucosterol with AChE will also further be verified by molecular docking as AChE inhibitor is of clinical use in AD. In PD-pathway, mostly inflammation- (for example, *COX2*) and apoptosis-related proteins (for example, *CASP3*, *CASP9*, and *CYCS*) were targeted by fucosterol (Figure 7B). On the other hand, in HD-pathway, proteins that are involved in antioxidant defense system (for example, *SOD1* and *GPX1*), neuronal growth and survival (for example, *BDNF*), inflammation (for example, *COX2*), and apoptosis (for example, *BAX*, *CASP3*, *CASP9*, *CASP8*, and *CYCS*) were the targets of fucosterol (Figure 7C).

### 2.6. Molecular Docking Simulation

We employed molecular docking analysis to validate the interaction patterns and efficiency of fucosterol to some of the vital target proteins, which are crucial regulators of several NDD-related molecular and cellular processes. Here we selected LXR-β, GR, TrkB, TLR2/4, BACE1, and AChE depending on their crucial roles in the inflammation, oxidative stress, apoptosis, and other risk factors associated with the pathobiology of NDD. Molecular docking followed by binding energy analysis showed that fucosterol showed the highest binding affinity to LXR-β, followed by GR and TrkB with binding energies of −80.37, −49.53, and −34.06 kcal/mol (Table S3), respectively, indicating that these receptor proteins are vital for the neuropharmacological activity of fucosterol. TLR2 also interacted with fucosterol with a binding energy of −33.86 kcal/mol. However, fucosterol showed a moderate binding affinity with other protein targets. We, therefore, considered LXR-β, GR, and TrkB for in detail binding interactions analysis by molecular dynamics simulation.

**Figure 8:**
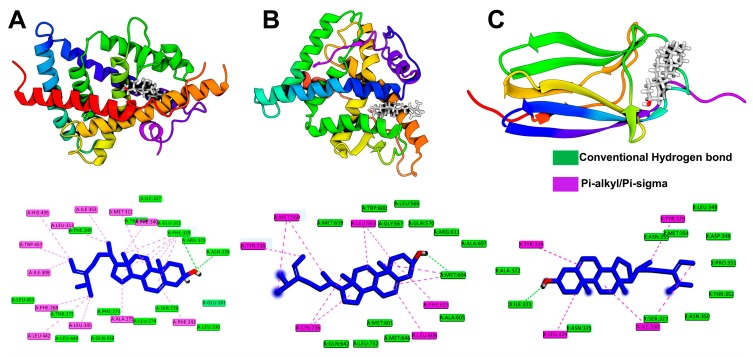
Binding and interaction pattern of fucosterol with the ligand binding domain (LBD) domain of LXR-β receptor (**A**), glucocorticoid receptor (**B**), and the extracellular domain of TrkB (**C**). The lower panel displays the corresponding two-dimensional representation of binding interactions occurred in the respective complex. The fucosterol made stable complex through hydrogen bonding (green) and the hydrophobic interactions (pink).

As an initial consideration, binding and interaction patterns revealed from molecular docking simulation were assessed. The result demonstrated that fucosterol formed significant interactions with these receptors following hydrophobic interactions. As shown in Figure 8A, fucosterol formed several hydrophobic interactions with LXR-β through alkyl and pi-alkyl bondings with active site residues, where the maximum interactions were formed with Phe329, including hydrogen bond with the hydroxyl group. The side chain of fucosterol also formed hydrophobic interactions with Leu313, Ile353, Leu345, Leu442, Phe268, Ile309, His435, and Trp457, respectively. The hydroxyl group also formed a hydrogen bond with Asn239, while the steroidal rings showed pi-alkyl interactions with Ala275, Met312, Phe329, and Phe243, respectively. Fucosterol exhibited similar binding behavior with GR, where maximum interactions were hydrophobic. The residues, including Met604, Leu563, Leu608, Cys736, Met560, Phe623, and Tyr735 formed non-bonded interactions with the fucosterol, where the steroidal ring displayed hydrophobic interactions with Leu563, Phe623, Leu608, and Met604, and the side chain with Tyr735, and Met560 (Figure 8B). The residue Met604 also formed hydrogen bonds with the hydroxyl group of fucosterol. In the case of TrkB, the residues Ile323, Leu324, Tyr329, Ile330, and Tyr319 were involved in making interactions with the fucosterol. All residues except Ile323 formed hydrophobic interactions, while the later formed hydrogen bonding to the hydroxyl group of fucosterol (Figure 8C).

### 2.7. Molecular Dynamics Simulation

**Figure 9:**
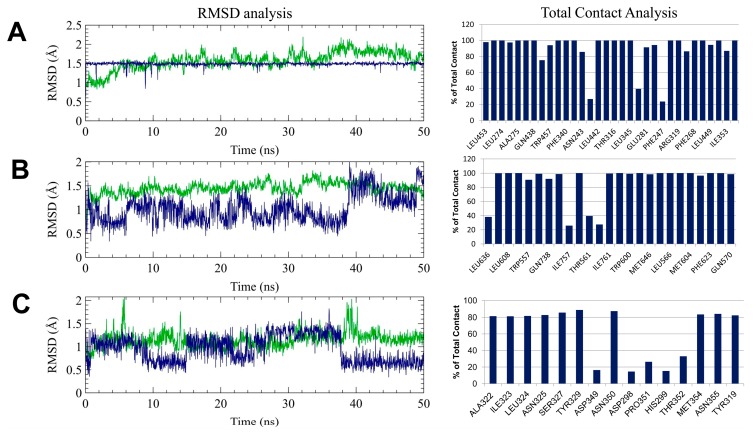
The analysis from 50 ns molecular dynamics simulations highlighting the conformational stability through root means standard deviation (RMSD; right panel) and total intermolecular contact (left panel) analysis for LXR-β (**A**), glucocorticoid receptor (**B**), and TrkB (**C**), respectively. The line plot shown in RMSD graph described the degree of flexibility for both protein (green) and ligand (blue) during the simulation.

The findings from molecular docking studies were further validated by 50 ns additional molecular dynamics simulations. Initially, the root means standard deviation (RMSD) of both protein and ligand from individual complexes was calculated by comparing with their initial position and represented in Figure 9A. The protein RMSD represents that all complexes achieved equilibrium during the 50 ns of simulation, where LXR-β obtained after 10 ns, GR and TrkB showed stability following 5 ns, respectively, concluding that the resulting trajectory is an appropriate basis for further analysis [33,34,35,36]. Furthermore, the ligand RMSD, which denotes the degree of ligand flexibility, described that fucosterol remained stable in the active site of LXR-β over the time of simulation, while it was seen to be flexible in the binding pocket of the GR and TrkB. Since fucosterol binds with these receptors by hydrophobic interaction, as revealed through molecular docking simulation, total contact analysis together with hydrogen bonding occupancy was further considered to solve the question whether fucosterol maintained similar interactions in the thermodynamics condition or not.

Interestingly, fucosterol maintained contacts with the active site residues, including Ile353, Leu345, Leu442, Phe268, Trp457, Ala275, Phe340, and Phe243 residues over the 80% of total simulation time, which were consistent with molecular docking simulation. In addition, fucosterol also showed maximum interactions with Arg319 and Glu281 residues during the simulation. Consequently, Met604, Leu608, and Phe623 residues in GR showed maximum contacts with fucosterol, while TrkB maintained hydrophobic interactions through the Ile323, Leu324, and Tyr329 residues. Furthermore, hydrogen bond occupancy showed that the hydroxyl group of fucosterol maintained hydrogen bond with Glu281 of LXR-β during the 10.74% of total simulation time but not with Asn239, which concluded that the conformational drift that exerted by fucosterol at the initial stage of simulation, changed the binding orientations, which might be more favorable and stronger than the complex predicted from docking simulation.

Physiologically, the activation of human LXR by either sterol and nonsterol agonists is usually exerted by a perpendicular histidine-tryptophan switch in the ligand binding domain, which turns the receptor into its active conformation, where the sterol guided activation is mediated by Trp457 [37]. Having sterol skeleton, fucosterol, and other phytosterols can bind in the ligand binding domain of LXR-β in a similar orientation as endogenous sterol ligands like estradiol, progesterone, and dexamethasone with their respective receptors such as estrogen, progesterone, and glucocorticoid receptors do [37,38]. This orientation was ensured by the non-bonded interaction with Glu281 [38], as also revealed by the present molecular simulation studies. Thus, our study concluded that the non-bonded interaction of fucosterol with Trp457 is the critical mechanism of the LXR-β activation.

The sifting of interaction pattern from Arg319 in molecular docking to Glu281 in molecular dynamics simulation suggests that fucosterol might induce conformational changes and thus activates LXR-β. In the case of GR, fucosterol showed a similar hydrogen bonding profile, where the compound maintained 8.94% of hydrogen bonding occupancy with Gln570; however, the hydrogen bonding pattern remained stable for TrkB, where Ile323 made 9.55% of H-bonding during the simulation. Together, it can be concluded that hydrophobic interactions play a significant role in the binding of fucosterol to LXR-β, GR and TrkB.

Several earlier reports highlighted that the fifth subdomain at the extracellular part of the TrkB receptor plays major role in the BDNF mediated TrkB interactions [39,40,41,42]. This domain furnishes a small molecule binding site [43]. The binding site is hydrophobic, where the residues Asn350, Pro351, Thr352, and Met354 directly involved in contact with BDNF [39]. In the case of GR, previous reports showed that the residues, including Trp557, Gln570, and Met604 play an essential role in ligand recognition and agonistic activity [44,45,46]. Similarly, molecular dynamics simulation followed by molecular docking studies showed that fucosterol maintained non-bonded interaction with these residues for more than 50 percent of total simulation time, supporting the fucosterol derived activation of GR signaling pathway.

## 3. Discussion

Here, we unraveled the molecular mechanism of neuropharmacological action of fucosterol against neurodegenerative disorders using integrated system pharmacology and molecular simulation approach. Initially, we characterized the pharmacokinetic behaviors of fucosterol, which supports its drug-like properties and accessibility to the brain tissue. Moreover, referring to its cholesterol-like chemistry, fucosterol could pass through the cell membrane to reach directly to various intracellular targets. The network pharmacology data indicated that fucosterol showed a close association with the target proteins of many crucial pathways at the molecular and cellular levels. Gene ontology-based bioinformatics analysis identified several enriched biological processes, including inflammatory response, apoptotic process, transcription regulation, aging, NF-κB activity, and cellular response to ROS, which are highly linked with the pathobiology of NDD. KEGG pathway analysis further demonstrates that fucosterol targeted proteins are the critical components of these highly enriched molecular and cellular pathways.

In the pathway analysis, inflammation-related pathways, including TNF, HIF-1, NF-κB, and VEGF signaling, are particularly enriched, indicating that the anti-inflammatory action of fucosterol could play a significant role in the treatment of NDD. Being a ubiquitous transcription factor that plays a crucial role in amplifying inflammatory and immune responses [47], NF-κB plays a central role in the pathogenesis of NDD. Aberrant regulation of NF-κB signaling in glial and immune cells propagates the pathological inflammatory response to the neuron, leading to neurodegeneration. The previous study reported that fucosterol attenuates inflammation through inhibiting NF-κB in LPS-induced RAW 264.7 macrophages [47], supporting the implication of fucosterol as an anti-inflammatory agent. Moreover, LXRs have been implicated in the immune and inflammatory responses in microglia. Originally, LXRs are cholesterol-sensing nuclear receptors whose primary function is to regulate cholesterol homeostasis in the brain. However, upon ligand activation, LXRs antagonize the expression of proinflammatory cytokines such as TNFα and IL-1β [48] as well as inflammatory genes such as NF-κB. Although there is currently no experimental evidence supporting the LXR-dependent anti-inflammatory effect of fucosterol, our *in silico* data showing a significant binding affinity of fucosterol to LXR-β suggest the involvement of a similar mechanism. Moreover, the glucocorticoid receptor (GR), upon ligand binding, exerts its potent anti-inflammatory actions through repressing NF-κB signaling [49]. The present molecular simulation data showing a significant binding affinity to GR also suggest an anti-inflammatory action of fucosterol.

Given the key role of cholesterol in brain physiology and function, disturbances in cholesterol homeostasis provoked inflammation and oxidative stress, and thus, have been associated with the onset of major NDDs [50]. LXRs, particularly LXR-β, play a critical role in brain cholesterol homeostasis. Upon ligand activation, LXR-β upregulates the expression of *ApoE*, *ABCA1*, *ABCG1*, and *SREBF1*, the key genes in reverse cholesterol transport [51]. The activity of ABC transporters mediates the cholesterol efflux to ApoE, which enhances Aβ clearance to blood [51,52]. Hoang and colleagues proved that fucosterol is a selective LXR-β agonist and that it regulates cholesterol homeostasis in multiple cells, including macrophages (functionally analogous to brain resident macrophage, microglia) through transcriptional activation of *LXR* target genes, such as *ABCA1*, *ABCG1,* and *ApoE* [17]. A similar study also reported the LXR-β -agonizing capacity of fucosterol in a luciferase reporter assay [53]. These findings suggest that fucosterol might produce a similar LXR-β-mediated effect in the brain. Moreover, as mentioned earlier, fucosterol showed the highest binding affinity to LXR-β, indicating that it could be a potential LXR-β agonist, which might play a significant role against AD pathology through maintaining cholesterol homeostasis and Aβ clearance involving ABC/SHREBF1/ApoE-dependent pathways.

Another enriched signaling pathway is the PI3K/Akt pathway, which, along with MAPK signaling pathway, regulates cellular growth and survival. This pathway has multiple downstream effectors, including those involved in cell survival (CREB, Bcl-2, Casp9, IKK, and NF-κB; Figure 4D). Our system pharmacology analysis showed that fucosterol targets CREB, which is a transcription regulator sensing the upstream signal from PI3K/Akt pathway, maintaining cell survival through upregulating Bcl-2. 

KEGG pathway analysis also demonstrates that nervous system-related pathways, including neurotrophin signaling and cholinergic, dopaminergic, and serotonergic synapses were enriched, suggesting that fucosterol might play a significant role in neuronal growth, survival, and functionality. Neurotrophin signaling pathway plays a crucial role in the maturation of developing neurons as well as in maintaining the growth and survival of adult neurons. Therefore, the deregulation of this pathway is evident to be associated with the NDD pathology. Insufficient neurotrophic support, further, suggests that neurotrophin mimetic could have therapeutic significance in the treatment of AD. Fucosterol targets TrkB, a classical receptor of brain-derived neurotrophic factor (BDNF), which suggests that this compound might function as BDNF-mimetic. Moreover, PI3K/Akt signaling pathway downstream to the neurotrophin signaling pathway was one of the enriched pathways in our study, indicating that these pathways might be employed in the pharmacological actions of fucosterol against NDD. In addition, Oh and colleagues reported that fucosterol protects against Aβ1-42-induced cytotoxicity through activating TrkB-mediated ERK1/2 signaling in primary hippocampal neurons, a TrkB-dependent neuroprotective effect, which was reversed by a selective TrkB inhibitor, cyclotraxin B, indicating its physiological interaction with TrkB in neuronal cells [30]. These in vitro cellular effects of fucosterol were further translated into in vivo effects, in which fucosterol ameliorates Aβ1-42-induced cognitive impairment in aging rats [30]. Since TrkB regulates major signaling pathways involved in neuronal growth and survival, we carried out *in silico* analysis with this protein target. Molecular docking followed by binding energy calculation revealed that fucosterol showed a significant binding affinity to TrkB, which indicates that fucosterol could function as BDNF-mimetic and modulate neuronal growth and survival through modulating the classical neurotrophin/PI3K/Akt signaling pathway. Furthermore, co-activation of TrkB/PI3K/Akt signaling pathway and Nrf2/ARE antioxidant system might synergistically confer neuroprotection against NDD pathology [54,55]. A previous report demonstrating the attenuating role of fucosterol against oxidative stress through upregulating antioxidant enzymes such as GPX1, SOD, CAT, and HO-1 via Nrf2 activation [7] and our *in silico* data on TrkB binding further suggest this possibility. 

Moreover, some vital synaptic proteins, including GluN2A and AChE were connected to fucosterol. GluN2A at the glutamatergic synapse mediates Ca^2+^ signaling, and thus plays a significant role in synaptic plasticity, which constitutes the biochemical basis of memory and cognition. The agonistic behavior of fucosterol to GluN2A could, therefore, help improve cognition deficits in AD patients. On the other hand, AChE catalyzes the breakdown reaction of acetylcholine at the cholinergic synapse, regulating synaptic transmission. However, AD pathology is accompanied by the cholinergic deficit, which could be compensated by the AChE inhibitor. Previous studies showing the inhibitory activity of fucosterol against AChE [12,28] and our molecular docking findings suggest that fucosterol could be a promising AChE inhibitor having clinical application in AD. 

In the KEGG pathway analysis, there was an enrichment of several immune-related pathways that are deregulated and compromised in NDD. In particular, several protein molecules of the TLR signaling pathway were targeted by fucosterol. TLRs play a critical role in innate immunity by recognizing pathogen-associated molecular patterns [56]. Deregulation of TLR signaling leads to the development of chronic diseases including NDD. Since TLRs play a significant role in the cellular immune response and its modulation can ameliorate inflammation, we carried out an *in silico* analysis with this protein target. Molecular docking analysis demonstrates that fucosterol interacted with both TLR2 and TLR4, suggesting that this compound could improve inflammation-induced NDD pathology through TLR-mediated immune response. In addition to their ability to activate the expression of genes linked to lipid metabolism, LXRs also antagonize inflammatory gene expression triggered by TLR activation [51].

β-Secretase cleaves amyloid precursor protein leading to the production of Aβ in the brain. Aberrant β-secretase activity is believed to be implicated in the pathogenesis of AD. Thus, targeting β-secretase is a viable therapeutic approach for AD. However, evidence suggests that the complete abolishment of β-secretase may be associated with specific behavioral and physiological alterations [57]. Therefore, natural products with reversible and non-competitive binding behavior have therapeutic promise against the β-secretase activity. Jung and his team reported that fucosterol shows noncompetitive inhibition against β-secretase [29]. Our network pharmacology and *in silico* analysis also revealed that fucosterol interacted with β-secretase, suggesting its potent anti-amyloidogenic activity.

## 4. Materials and Methods

### 4.1. ADME/T Analysis of Fucosterol

Pharmacokinetic parameters including absorption, distribution, metabolism, and excretion/transport (ADME/T) of fucosterol were analyzed through QikProp (Schrödinger Release 2019-3: QikProp, Schrödinger, LLC, New York, NY, USA). QikProp is an efficient ADME/T prediction tool that forecasts whether the selected compound would exhibit satisfactory ADME/T performances during clinical trials.

### 4.2. Data Mining for Target Selection

The information on fucosterol targets was retrieved primarily from PALM-IST database. We verified every target protein through the PubMed link. We excluded those targets that have no direct interaction with fucosterol and included additional targets while scanning PubMed database. The protein information, including protein name, gene ID, and host organism were verified through UniProt (http://www.uniprot.org/) [58].

### 4.3. Network Building

#### 4.3.1. Fucosterol–Target–Target (F–T–T) Network

A protein–protein interaction (PPI) network among the targets was built using Search Tool for the Retrieval of Interacting Genes/Proteins (STRING), an online tool for functional protein association networks. The fucosterol-target network and PPI network were merged through Cytoscape v3.7.1 (Seattle, WA, USA) [59]. This network consists of nodes and edges representing molecules (fucosterol and targets) and intermolecular interactions, respectively.

#### 4.3.2. Fucosterol–Target–NDD (F–T–NDD) Network

The lists of AD, PD, and HD-related genes were retrieved from DisGeNET database v6.0 (Barcelona, Spain) [60], a database that integrates human gene-disease associations from expert-curated databases and text-mining derived associations. The targets related to NDD are those that were matched with the targets of fucosterol. The active F–T–NDD networks were established by Cytoscape v3.7.1.

### 4.4. Gene Ontology (GO) Analysis

The functional enrichment analysis of GO for the biological process, molecular function, and cellular components was performed and presented in bar graphs using Network Analyst [61] (https://www.networkanalyst.ca/). GO terms with a *p*-value of < 0.05 were considered significant. 

### 4.5. Network Pathway Analysis

Gene functional annotation for KEGG pathways was retrieved from the Database for Annotation, Visualization and Integrated Discovery (DAVID) v6.8. First, gene counts for each pathway in the given gene set were calculated. Then, significantly enriched pathways in the given gene set compared to the genome background are defined by a hypergeometric test. Taking *p* values < 0.05 as a threshold, pathways that meet this condition were defined as significantly enriched pathways in the given genes set. Enriched pathways were categorized using KEGG pathway database [62] (https://www.genome.jp/kegg/). Pathways highlighting fucosterol targets were retrieved through the KEGG pathway mapper [63] (https://www.genome.jp/kegg/tool/map_pathway2.html).

### 4.6. Molecular Docking and Binding Energy Analysis

The three dimensional crystal structure of human LXR-β (PDB ID: 1P8D), GR (PDB ID: 6DXK), TrkB (PDB ID: 1HCF), TLR2 (PDB ID: 6NIG), TLR4 (PDB ID: 5IJC), BACE1 (PDB ID: 5HDZ), and AChE (PDB ID: 3QT0) were retrieved from the protein data bank [64] and prepared for molecular docking studies by using protein preparation module of Schrödinger 2017-1 following previously described protocols [34,35,65,66,67,68]. Briefly, the amino acid orientation in the PDB file was fixed by correcting bond orders, adding charges and hydrogen. Following that the structure was optimized at neutral pH (7.0 ± 2.0), and then minimized by using OPLS 3 force filed limiting maximum heavy atom RMSD to 0.30 Å. The candidate compounds structures were prepared for molecular docking by minimizing with OPLS 3 force field through Ligprep2.5 in Schrödinger Suite (2017-1). The Epik2.2 module was used to generate the ionization state of each compound at pH 7.0 ± 2.0. For molecular docking analysis, the receptor grids were fixed at the ligand-binding site of the receptor. In both cases, a cubic box of specific dimensions centered on the centroid of residues involved in the ligand-binding site was generated. The bounding box was set to 18 Å × 18 Å × 18 Å for docking experiments, keeping default parameters of glide docking procedure. After that glide docking with an extra precision setting (XP) performed with default settings, consisting of Van der Waals scaling factor and partial charge cutoff for ligand atoms of 0.80 and 0.15, respectively. Binding free energy was calculated to rescore and for choosing the top hits from the candidate ligands. In the Prime MM-GBSA method, the calculation of binding energy was performed by combining OPLSAA molecular mechanics energies (EMM), an SGB solvation model for polar solvation (GSGB), and a non-polar solvation term (GNP) composed of the non-polar solvent accessible surface area and van der Waals interactions [69]. Here, as the source in Prime MM-GBSA simulation, the glide pose viewer file of the best conformation was given. For modeling directionality of the hydrogen-bond and π-stacking interactions, the dielectric solvent model VSGB 2.0 [70] was used to apply empirical corrections. Keeping the protein chain flexible [71,72,73,74,75], minimizing approach is applied as sampling methods. The analysis denotes more excellent binding by more negative binding energy. Overall free energy of binding:

ΔG_bind_ = G_complex_ − (G_protein_ + G_ligand_), where G = EMM + GSGB + GNP.

### 4.7. Molecular Dynamic Simulation

After that, the docked complexes were subjected to molecular dynamics simulations using Yet Another Scientific Artificial Reality Application (YASARA) v.16.9.23 (YASARA Biosciences GmbH) Dynamics software. Before the simulation, all structures were cleaned and subjected to the optimization of hydrogen bonding network. For each simulation system, a cubic simulation cell with periodic boundary condition was generated and then all atoms were parameterized with the Assisted Model Building with Energy Refinement (AMBER14) [76] force field. The transferable intermolecular potential 3 points (TIP3P) water model was used to make the solvation system, and the density was maintained to 0.997 gm/L. Using the simulated annealing method, the initial energy minimization process of each simulation system was done by using the steepest gradient approach for 5000 cycles. Molecular dynamics simulations were done by using the PME methods to describe long-range electrostatic interactions at a cut off distance of 8 Å at physiological conditions (298 K, pH 7.4, 0.9% NaCl) [77]. Multiple time-step algorithms along with a simulation time step interval of 2.50 fs were chosen [78]. At constant pressure and Berendsen thermostat, molecular dynamics simulations were performed for 100 ns long, and MD trajectories were saved every 25 ps for further analysis.

## 5. Conclusions

The network pharmacology and *in silico* findings demonstrate that fucosterol interacted with the proteins of major molecular and cellular pathways that are involved in neuronal growth and survival, neuroinflammation, and immune response, and thus could help modulate and improve pathobiology of neurodegeneration. Our system biology approach to unravel the neuropharmacological action mechanism of fucosterol further offers a platform to explain the complex pharmacological mechanism of a bioactive molecule against NDDs in a multitarget approach. However, further experimental validation by cellular and animal studies is warranted before its recommendation for clinical application.

## Supplementary Materials

The following are available online at www.mdpi.com/xxx/s1. Figure S1: Inflammatory pathways in KEGG pathway analysis, Table S1: ADME/T properties of fucosterol, Table S2: Data for the fucosterol targets used to construct F–T–T and F–T–NDD networks, Table S3: Molecular docking simulation findings of fucosterol targets.
