# Supplementary figures and images for: Deciphering Molecular Mechanism of the Neuropharmacological Action of Fucosterol through Integrated System Pharmacology and In Silico Analysis
Source: Mar Drugs. 2019 Nov 13;17(11):0. doi: 10.3390/md17110639 (PMC6891791; doi:10.3390/md17110639)

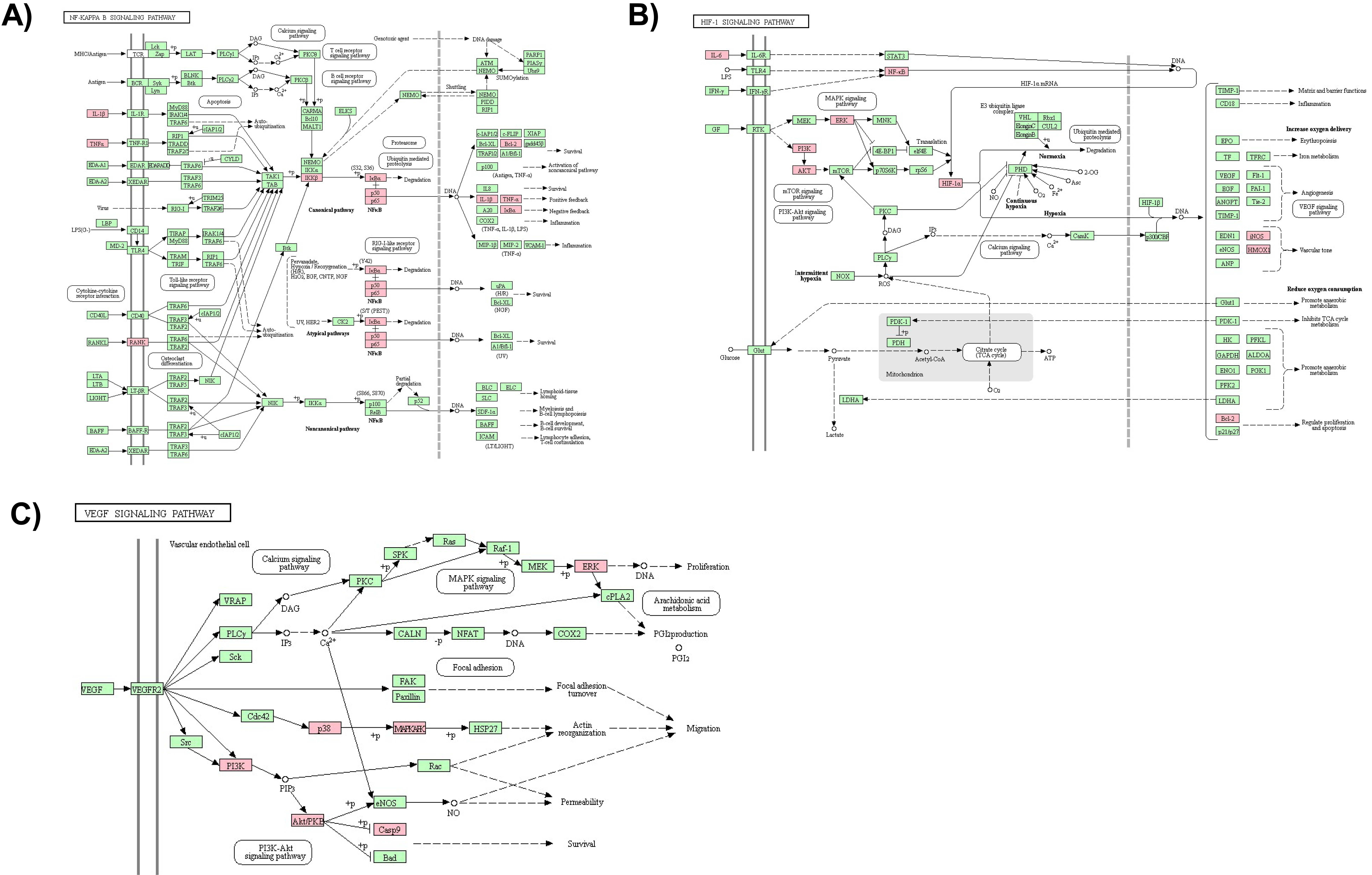

Supplement: Supplementary file 1 [file marinedrugs-17-00639-s001.zip › Supplementary files/Figure S1. Inflammatory pathway in KEGG pathway analysis.jpg]
